# Invasive Fungal Infections after Renal Transplantation

**Published:** 2012-02-01

**Authors:** S. Ezzatzadegan, S. Chen, J. R. Chapman

**Affiliations:** 1 *Shiraz Nephrology Urology Research Center, Shiraz University of Medical Sciences, Shiraz, Iran,*; 2 *Center for Infectious Diseases and Microbiology, Westmead Hospital , Westmead, Sydney , NSW, Australia,*; 3 *Center for Transplant and Renal Research, University of Sydney, Westmead Hospital, Sydney , NSW, Australia*

**Keywords:** Fungal infections, Renal transplantation, Cryptococcosis

## Abstract

Background: Invasive fungal infection (IFI) is a leading cause of infection-related mortality among kidney allograft recipients.

Objective: To estimate the incidence and etiology of systemic fungal infection in renal allograft recipients in Sydney transplant facility.

Methods: 471 kidney recipients, transplanted between 2000 and 2010 at the Westmead Hospital renal transplantation center, Sydney, Australia, were retrospectively surveyed.

Results: IFI developed in 10 (2.1%) of 471 patients. With a mean±SD new kidney transplants per year of 42.9±13, the mean±SD incidence of IFI was 0.9±0.6 for each year of transplantation. 4 patients had received kidneys from living donors and 7 from cadavers with a mean±SD age of 50.5±14 years. The mean time to IFI was 33 months after transplantation with majority within the first 2 years. *Cryptococcus neoformans *was responsible for 50% of episodes (n=5) followed by *Aspergillus fumigatus* (n=3), and *Pseudallescheria boydii* (n=3); there was a single case of mucurmycosis. Lungs (n=5) followed by meninges (n=4) and skin (n=3) were the most commonly involved sites.

Conclusion: IFI remains a major concern in renal transplantation. A high index of suspicion is required for early diagnosis and treatment to reduce the mortality. In this regard, appropriate diagnostic tests are necessary, particularly for *C. neoformans*.

## INTRODUCTION

Since kidney transplantation was first introduced, the lives of thousands of people with end-stage renal failure have been substantially prolonged. Breakthroughs in the field of immunosuppressive therapy have led to the improved survival of the grafts and the recipients. Inevitably, the incidence of opportunistic infections has increased as a consequence of this immune suppression. Other than bacterial and viral pathogens, fungi have had a major contribution to the high mortality and morbidity rate of opportunistic infections among kidney transplant recipients [1,2].

Among patients with solid organ transplantation, recipients of kidney transplants have been associated with the lowest rate of invasive fungal infection (IFI) (1%–14%) [1,3-5]. Since the incidence is apparently low, there are few reports of the epidemiology of IFI in this group of patients. Suppression of the immune system, particularly within one to six months after transplantation, results not only in increased propensity to fungal infections but also may mask the presentation of these infections which leads to delay in diagnosis [1,6]. Therefore, determining the risk factors, epidemiology and clinical manifestations as well as identifying the best diagnostic approaches and treatment plans are essential to inform appropriate management to minimize morbidity. 

In this study we report on our experience of IFI in kidney transplant recipients at a large transplant centre in Sydney, Australia, from 2000 to 2010.

## MATERIAL AND METHODS

We retrospectively reviewed the medical records and drug charts of 471 renal transplant recipients who were transplanted at Westmead Hospital, Sydney, Australia, from January 2000 to December 2010 for evidence of IFI. Of 471 recipients, 253 (53.7%) had received kidney from living donors and 219 (46.3%) from cadavers.

Clinical data collected included age, gender, donor (living or cadaver), age at transplantation and primary disease (Table 1). In addition, we gathered information on changes in immunosuppressant medications during infection and any rejection episodes and relevant treatment occurred prior to the infection.

**Table 1:** Features of 10 kidney allograft recipients with invasive fungal infection

**Patient No.**	**Age/Sex**	**Date of** **Transplantation**	**Donor**	**Underlying** **Disease**	**Immunosuppression**
1	26/F	2004	Living related	Diabetes mellitus	T, A, P
2	51/F	2006	Deceased	UNK	T, M, P
3	61/F	2004	Living unrelated (Friend)	ADPKD	C, M, P
4	58/M	2001	Deceased	IgA nephropathy	M, S, P
5	27M	2000	Living related	Glomerulonephritis	C, M, P
6	72/M	2009	DCD	IgA nephropathy	T, M, P
7	41/F	2008	Deceased	Dysplastic kidneys	C, M, P
8	46/F	2001	Deceased	Diabetes mellitus	C, A, P
9	55/M	2007	Living related	Diabetes mellitus	T, L, P
10	68/M	2010	Deceased	Glomerulonephritis	T, M, P

F: Female; M: Male; DCD: Donation after cardiac death; ADPKD: Autosomal dominant polycystic kidney disease; T: Tacrolimus; C: Cyclosporine; M: Mycophenolate mofetil; A: Azathioprine; P: Prednisolone; S: Sirolimus; L: Leflunomide; UNK: Unknown

In terms of fungal infection, we collected data for type of the isolated fungus and its species, timing after transplantation, clinical presentation, the organs involved (site of infection), all relevant microbiological and histological test results, imaging results, antifungal treatments and other management as well as follow-up and prognosis. We also looked for any concurrent viral and bacterial infections. 

Diagnosis of IFI was confirmed by a positive culture (tissue, blood, CSF and aspirated fluids), detection of antigens in blood and CSF, and direct histopathologic evaluation of tissue specimens (Table 2). Chest X-ray, tomography, brain CT and MRI-scans were done based on the signs and symptoms, to detect organ involvement and assess complications as well as the response to the treatment. Infections were classified as “definite” and “probable” IFI according to previously determined criteria [7]. According to our protocol, nystatin 4 mL/day for one month and trimethoprim-sulphamethoxazole (80/400 mg/day) for at least six months were used as prophylaxis in all kidney recipients.

**Table 2:** Features of invasive fungal infections in the kidney recipients

**Patient No./** **Episode No.**	**Timing after Transplant. (months)**	**Pathogen**	**Site**	**Presentation**	**Diagnostic procedure**	**Treatment**	**Outcome**
1/1	14	*Cryptococcus neoformans*	Lung, Meninx, Skin	Headache, fever, pleuritic pain, lethargy	BAL/C, blood PCR, inner nose tissue/C	Liposomal ampho B, fluconazole	Remission
1/2	21	*Neosartorya fisheri*	Lung	Headache, fever, cough	Lung abscess/C	L-ampho B, voriconazole, caspofungin, posaconazole	Died of cryptococcal meningitis sequel
2/1	15	*C. neoformans*	Meninx	Headache, myalgia, obtundation	CSF PCR/C	L-ampho B, 5-flucytosin, Fluconazole	Died
3/1	21	*C. neoformans*	Lung	Asymptomatic	BAL/C, blood PCR	L-ampho B, 5-flucytosin, fluconazole	Remission
4/1	88	*C. neoformans*	Meninx	Fever, headache, nausea/vomiting	CSF/C	L-ampho B, 5-flucytosin, fluconazole	Remission
5/1	117	*C. neoformans*	Lung, Meninx	Productive cough and fever for 3 months	Blood & CSF antigen, BAL/C	L-ampho B, 5-flucytosin, fluconazole	Remission
6/1	5	*Aspergillus fumigatus*	Chest	8 months of multiple painless nodules on the leg, buttock and chest wall	Chest FNA/C	Caspofungin, voriconazole	Remission, died of sepsis later
7/1	23	*A. fumigatus*	Lung	Intermittent dry cough for 6 months	BAL smear	Data unavailable	Follow-up data unavailable
8 /1	66	*Mucor *spp.	Sinus	Sinusitis	Tissue biopsy	L-ampho B, posaconazole	Remission
9/1	6	*Scedosporium apiospermum *	Skin	Skin nodules & pustules on fingers	Culture of aspiration	Voriconazole	Remission
9/2	17	*S. apiospermum *	Spine, Aorta	Bilateral loin & back pain for 6 months	Culture of abscess aspiration + tissue culture	Caspofungin, voriconazole	Died of bowel infarction
10/1	5	*S. apisopermum* *Exserohilum* spp.	Skin Sinus	Left forearm nodules Headache for 2 months	Skin/C Sinus tissue/C	Voriconazole	Remission

BAL: Bronchoalveolar lavage; C: Culture; CSF: Cerebrospinal fluid

## RESULTS

Demographics and incidence 

Diagnosis of IFI was made in 10 (2.1%, 95% CI: 0.8%–3.4%) of 471 renal transplant recipients. With a mean±SD new kidney transplants per year of 42.9±13, the mean±SD incidence of IFI was 0.9±0.6 for each year of transplantation. All patients had proven fungal infection [7]. There were five women and five men involved. The mean±SD age of studied patients was 50.5±14 (range: 26–72) years, with six (60%) over 50 and two (20%) less than 30 years. Four of the patients had received allograft from living donors and seven from cadaver (Table 1). All studied patients have been transplanted in our center.

Patient characteristics and predisposing factors 

The mean±SD time to diagnosis of IFI was 33.1±35 months (range: 5 m to 10 y) after transplantation, mostly within first two years (n=7) (Table 2). Of nine patients with known HLA status, six had more than three mismatches with the donors. Evidence of interstitial fibrosis/tubular atrophy associated with abnormal serum creatinine was present in four patients. Worsening of allograft function occurred in three patients during treatment of fungal infection. No significant abnormality was seen in serum electrolytes. Of 10 patients with IFI, eight were on triple immunosuppressive therapy with calcineurin inhibitor (4 tacrolimus and 4 cyclosporine), antimetabolites (2 azathioprine, others mycophenolate) and prednisolone. Of the remaining two patients, one was on sirolimus, mycophenolate and prednisolone, the other one received tacrolimus, leflunomide and prednisolone (for BK nephropathy) (Table 2). 

Antimetabolites were discontinued or reduced in six patients due to severity of the disease. Rejection occurred in five cases within the preceding year of the fungal infection, two of them with two episodes of rejection, one of which required OKT3 for vascular rejection. Three pulses of 500 mg methylprednisolone was given to all clinically significant rejections.

Patients’ files and charts were also reviewed for any concurrent viral infection. No CMV infection had been reported. There was just a case of BK associated nephropathy at the same time with fungal infection.

Etiology of IFI 

There were 12 episodes of IFI in 10 patients with two patients experiencing two separate episodes, 7 and 11 months apart, respectively. The most common IFI was cryptococcosis (n=5) followed by invasive aspergillosis (n=3), scedosporiosis (n=3), and a single cases of mucurmycosis. More than one organ was involved in four of 12 IFI episodes; lung (n=5), meninges (n=4) followed by skin (n=3), sinus (n=2), spine (n=1) and aorta (n=1) (Table 2) were the most common sites of involvement. Some patients had non-specific symptoms for up to three months prior to diagnosis. In a case of cryptococcosis, no symptoms were reported and the suspicion was based on abnormal chest X-ray findings. In patients with lung involvement, the most frequent abnormalities were nodules and cavities, in contrast to meningitis with no specific abnormalities in nearly all cases. Headache was the most common complaint in meningitis associated with fever in most cases.

*Cryptococcus neoformans *was isolated in five patients, two with meningitis, one with lung involvement and another two cases with simultaneous meningitis and lung involvement (Table 2). Diagnosis of cryptococcosis was made by culture of CSF and/or bronchoalveolar lavage (BAL) or lung abscess fluid and by cryptococcal antigen detection in CSF and in serum. The patients were commenced on combined amphotericin B (liposomal) and 5-flucytosine for at least six weeks and thereafter on oral fluconazole. One patient with cryptococcal meningitis developed a lung and cerebral abscess caused by *Neosartorya fischeri* seven months later. Due to slow response to treatment, lobectomy was done. Unfortunately, after operation, repeated tonic-clonic seizures ensued, resulting in death.

*Aspergillosis fumigatus* infection was reported in two patients—one with lung parenchymal involvement, and the other with chest wall abscess. Diagnosis was made by recovery of the organism from BAL and abscess, respectively.

Mucurmycosis was found in one patient who had presented with symptoms of sinusitis and mucosal thickening of maxillary sinuses. Initially, she had been treated as bacterial sinusitis. The fungus was revealed on histopathology of the mucosal biopsy.

Skin nodules and pustules on extremities were the main presentations in the two patients with *Pseudallescheria boydii/Scedosporium apiospermum* infection, diagnosed by culture of the fluid aspirated from the pustules (Table 2). The following year, one of the patients (patient no. 9) presented with bilateral back pain of six months duration. Osteomyelitis of the lumbar vertebra with abscess formation followed by aneurysmal dilation of abdominal aorta due to Scedosporium aortitis [8]. The other patient had *S. apiospermum* grown from skin nodules and also suffered from invasive sinusitis involving the frontal, mastoid bone and ethmoid sinuses caused by *Exserohilum spp*. (Table 2).

Treatment 

For treatment of cryptococcosis, the patients were commenced on combined amphotericin B (liposomal) and 5-flucytosine for at two to six weeks and then on oral fluconazole (Table 2). The time for cryptococcal antigen to become negative ranged from three months to as long as three and a half years. 

Voriconazole, caspofungin, liposomal amphotericin B and posaconazole, either as single agents or in combination, were used to treat invasive aspergillosis. Voriconazole was also the main antifungal agent used in the treatment of scedosporiosis which was combined with caspofungin in patient no. 9 (Table 2). In the patient with mucurmycosis, liposomal amphotericin B was started but was switched to posaconazole after eight days due to a rise in serum creatinine.

## DISCUSSION

Over recent years, with introducing new immunosuppressive strategies, graft survival of kidney transplant recipients has significantly improved but inevitably has led opportunistic pathogens to play a major role in the high mortality of infections in these patients [5]. The variable reported incidence of fungal infections can be explained by interaction between environmental factors, state of immunosuppression and the availability of diagnostic measures.

The earliest studies reported significantly high incidence of fungal infections of about 45% in kidney recipients [9]. Later reports showed declining rates with the lowest incidence of 1.5% [10,11].

Environmental factors including hygiene status and sanitary conditions have resulted in higher rates of fungal infections in developing countries, while it is believed that these rates could be higher in the absence of appropriate diagnostic measures in these countries [9]. The etiology of IFI may also differ between developed and developing countries. For example, cryptococcosis is more common in the studies from developed countries [10,12] in contrast to developing countries where this infection is rare [1,3,4]. Our reported incidence of 2.3% is consistent with the western countries’ studies but this rate is still an area of concern. Despite the good hygiene conditions in Australia, using more potent immunosuppressive drugs, tacrolimus and mycophenolate instead of cyclosporine and azathioprine, could result in the persistence of fungal infections. On the other hand, thanks to applying appropriate diagnostic investigations, infections are detected and then treated earlier.

Older age, diabetes mellitus, CMV infection, allograft dysfunction and treatment of rejection have been reported as predisposing factors for IFI [1,3,4]. Although 63% of our patients were above 50, IFI occurred in two patients less than 30 years old. Diabetes mellitus was detected as the underlying disease in four patients. None of the fungal infections was associated with CMV infection in our report; it is likely due to appropriate prophylactic regimen of 3 to 12 months.

While nearly half of our cases comprised cryptococcosis, death occurred in four patients which is lower than reported mortality of 50% to 75% in other studies [1,3,4]. None of the deaths was in the acute phase of infection and all were due to the late complications of the infection (Table 2). Using appropriate anti-fungal agents such as liposomal amphotericin, flucytosine, caspofungin and voriconazole along with close and careful follow-up and long-term oral fluconazole, particularly in patients with cryptococcosis, have resulted in lower mortality rate.

Invasive candidiasis has been reported as the most common IFI in the vast majority of epidemiological reports [4,12,13]. Administration of oral nystatin, as prophylaxis, for at least three months and universal prophylaxis of CMV infection, which is associated with candidiasis [4], could explain the lack of candidemia or other forms of invasive candidiasis in our study.

*P. boydii/S. apiospermum* is a fungus found in water and soil [14]. Typical manifestations include skin/subcutaneous involvement, brain abscess and lung disease. Systemic infection is rare in an immunocompetent host but may occur in organ transplant patients affecting the sinuses, lungs, bones and central nervous system [14-16]. The patients described herein first presented with skin nodules and pustules, with late complications of bone and aortic aneurysm seen in one of them. The genus Scedosporium has two clinically important species: *Scedosporium apiospermum* (*Pseudallescheria boydii*) and* S. prolificans. S. prolificansis *is more virulent than* S. apiospermum *and associated with more fungemia and higher mortality rate in solid organ transplant recipients [17]. The recommended treatment is voriconazole [18].

Mucurmycosis has been reported as a less common cause of IFI in renal transplantation. There is just one study from Iran in which it is the most common form of IFI [3].

Cryptococcosis is a fungus more commonly reported in kidney transplant recipients other than other solid organ recipients [12]. In contrast to some previous reports, it was the most commonly observed IFI in our survey. Distinct geographic regions may be associated with greater risk for cryptococcosis although the infection is most closely associated with underlying host risk factors such as HIV/AIDS and immune suppression from organ transplantation. Infection has also been epidemiologically associated with exposure to pigeon guano and eucalypt trees, although links to human infection remain circumstantial [19]. Although it is widely accepted as a reactivation of a latent infection, there are some studies about the primary origin of the cryptococcosis in transplant recipients [20-22]. With unknown reasons, it has been rarely seen within the six months of transplantation [4,12]. Similar to our findings, Cryptococcal meningitis usually presents with headache, fever and nausea/vomiting with unremarkable brain imaging results [19]. As we have also found, pulmonary involvement usually manifests with non-specific symptoms and even asymptomatic radiologic findings [23]. Papular, nodular and ulcerative lesions are manifestations of cutaneous cryptococcosis [24]. Although cutaneous lesions can be primary, it is important to know that disseminated cryptococcosis may present initially as skin lesions; therefore, early biopsy of skin lesions is recommended [4,22]. It has been shown that cryptococcosis is more likely to present as cutaneous infection than central nervous system involvement in patients who are on calcineurin inhibitors [25]. Culture, direct microscopic examination and detection of antigen are the ways to diagnosis [2]. In meningitis, the sensitivity of India ink test is 50%–80% and antigen over 90%–100% and it is essential to know that antigenemia could be undetectable in isolated pulmonary involvement [2,24]. The treatment of invasive central nervous system cryptococcosis is induction therapy with amphotericin B together with flucytosine for at least 14 days, followed by oral fluconazole for at least 6–12 months. Lipid formulations of amphotericin are preferred as they are better tolerated particularly in those with some degrees of renal impairment [24].

Kidney transplant recipients have the lowest risk of aspergillosis among solid organ transplants [2]. Although a study from Turkey identified it as the most common IFI, in majority of reports it is second to candidiasis in majority of reports [1]. Similar to our patients, lung is the most prominent site of infection. Fever, dyspnea and dry cough are usual presentations. Tomography is preferred to simple radiography if aspergillosis is suspected. Radiographic findings include focal infiltrations, nodules and cavities [2]. Voriconazole is now the drug of choice and preferred to amphotericin [26]. Documentation of diagnosis is by identification of hypha invasion or culture of the involved tissue [2].

In conclusion, IFI continues to be a major challenge in the field of transplantation. Early diagnosis and prompt treatment are essential in reducing the mortality rate. Diagnosis of fungal agents such as Cryptococcus is needed to be focused further, as there are no appropriate tests available in most transplant centers, particularly in developing countries. Furthermore, regarding the ever-changing nature of fungal infections, it is important to re-evaluate the epidemiology of IFI in transplant recipients, even in centers where such studies have already been performed in the past.
